# Joint association of weight-adjusted-waist index and physical activity with insulin resistance in adolescents: a cross-sectional study

**DOI:** 10.1186/s12902-024-01633-1

**Published:** 2024-07-01

**Authors:** Yong Zhou, Peng Tang, Yican Wang, Ying Tang, Yujian Yang

**Affiliations:** 1 School of Public Health, Xiangnan University, Chenzhou, Hunan 423000 China; 2 Department of Epidemiology and Health Statistics, School of Public Health, Guangxi Medical University, Nanning, 530021 China; 3 Department of Occupational Health and Environmental Health, School of Public Health, Anhui Medical University, No. 81 Meishan Road Hefei 230000, Hefei, Anhui 230000 China; 4 Department of Thoracic Surgery, Changsha Hospital of Traditional Chinese Medicine, Hunan University of Traditional Chinese Medicine, No. 22 Xingsha Road, Changsha, Hunan 410100 China

**Keywords:** Cross-sectional study, Homeostasis model assessment of insulin resistance, National Health and Nutrition Examination Survey, Physical activity, Weight-adjusted waist index

## Abstract

**Background:**

The weight-adjusted waist index (WWI) is a recently developed obesity metric, and the aim of this study was to investigate the relationship between physical activity (PA) and WWI and the homeostasis model assessment of insulin resistance (HOMA-IR) in adolescents, as well as the joint association of HOMA-IR.

**Methods:**

This study was based on the National Health and Nutrition Survey conducted between 2013 and 2016 and included 1024 adolescents whose median age was 15.4. Multivariate linear regression was used to examine the associations between HOMA-IR and PA and WWI. Using generalized additive models, a potential nonlinear link between WWI and HOMA-IR was evaluated. Subgroup analysis was also carried out.

**Results:**

The fully adjusted model revealed a positive association (β: 0.48, 95% CI: 0.43, 0.53) between the WWI and HOMA-IR. The HOMA-IR was lower in physically active (β: -0.16, 95% CI: -0.26, -0.05) participants versus inactive participants. Participants who had higher WWI and were not physically active (β: 0.69; 95% CI: 0.56, 0.82) had the highest levels of HOMA-IR compared to participants who had lower WWI and were physically active. Subgroup analysis revealed that these correlations were similar in males and females.

**Conclusion:**

Our results demonstrated that higher WWI and PA were associated with a lower HOMA-IR and that WWI and PA had a combined association with HOMA-IR. The findings of this study are informative for the preventing insulin resistance in adolescents.

**Supplementary Information:**

The online version contains supplementary material available at 10.1186/s12902-024-01633-1.

## Introduction

Insulin resistance (IR) is a clinical condition characterized by a decrease in glucose uptake and utilization by target cells, leading to a compensatory increase in insulin secretion, which triggers chronic hyperinsulinemia [1]. Diabetes, hypertension, obesity, dyslipidemia, and other metabolic and cardiovascular changes have been associated with IR, which, in addition to being a component of metabolic syndrome, constitutes an independent risk factor for cardiovascular disease [2]. In addition, IR is linked to chronic inflammation, which accelerates the development of atherosclerosis by promoting the growth of vascular smooth muscle cells, increasing the rate at which collagen is formed, and producing an excessive amount of growth factors [3]. Increased IR in adolescents increases the risk of obesity, type 2 diabetes mellitus, and coronary heart disease in adulthood [1, 4].

Obesity is defined as an abnormal or excessive amount of body fat that adversely affects health and is strongly associated with the development of several chronic diseases [5]. In the US, approximately 14.4 million children and adolescents are affected by childhood obesity, which is a serious concern [6]. Common measurements used to evaluate obesity include body mass index (BMI) and waist circumference (WC). However, in recent years, concerns have been raised about the accuracy of BMI [7, 8]. The BMI is commonly used to assess and classify obesity. However, it cannot distinguish between central and peripheral fat or between body fat and lean body mass. According to recent studies, body composition and fat distribution can be used to more accurately identify metabolic problems [9, 10]. The weight-adjusted waist circumference index (WWI) is a new obesity index that standardizes WC to body weight and combines the advantages of waist circumference [11]. In addition to distinguishing between muscle and fat mass, the WWI also reflects central obesity issues independent of body weight [12]. Numerous studies have demonstrated that WWI is more accurate than BMI [13, 14]. As a new clinical indicator, the WWI has the potential to improve the accuracy of obesity classification and risk prediction, thereby informing more targeted therapeutic interventions and monitoring strategies. However, the relationship between WWI and IR remains unexplored.

Physical activity (PA) is an activity that promotes health, strengthens physical fitness and enriches life through a variety of physical activities according to the needs of the body [15]. Numerous studies have shown that exercise interventions can improve metabolic parameters, such as lipid profiles, IR indicators, and other related hormones, in adult populations [16]. Exercise training has been shown to play a role in reducing IR in youth [17, 18]. Physical activity is linked to improved insulin sensitivity in childhood, which may have long-term effects on beta-cell health and the risk of developing diabetes in the future [19].

Physical activity and weight loss are frequently shown to improve IR in diabetic patients as well as to delay or prevent the onset of the disease in those who are at risk of getting it [20]. Since both obesity and PA can individually affect HOMA-IR in different direction, whether WWI and PA have combined association with HOMA-IR are still not clear. Hence, the purpose of this research was to investigate the correlation between WWI and physical activity with homeostasis model assessment of insulin resistance (HOMA-IR), and the combined association of WWI and PA with HOMA-IR in US adolescents aged 12–19 years.

## Method

### Study population

**Fig. 1:**
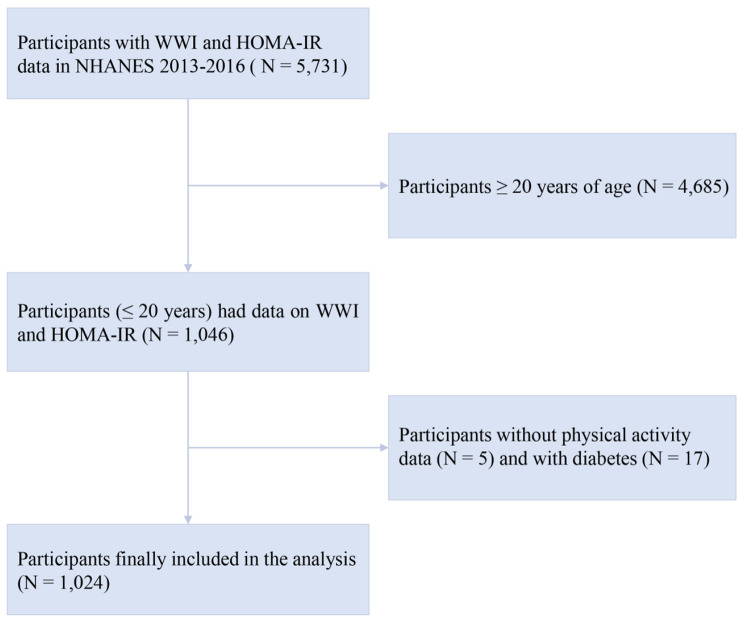
Flowchart of participant selection

The data for this study were obtained from the National Health and Nutrition Examination Survey (NHANES) [21], which was conducted by the National Center for Health Statistics (NCHS) from two cycles (2013–2014 and 2015–2016). The NHANES is an annual cross-sectional survey that estimates and assesses the health, nutritional status, and potential risk factors of noninstitutionalized civilians in the United States. The study protocol was approved by the Research Ethics Review Board of the NCHS. All subjects gave informed written consent before to participation. In the beginning, a total of 5,731 participants were enrolled in the study with complete information on both the WWI and HOMA-IR. Following elimination for age ≥ 20 years (*n* = 4,685), missing data on physical activity (*n* = 5). Patients with diagnosed diabetes (*n* = 17) were further excluded in order to avoid the impact of diabetes therapy on relevant serum markers. Diabetes was defined as a glycated hemoglobin ≥ 6.5%, a fasting plasma glucose ≥ 126 mg/dL, self-reported diabetes, or current use of insulin or diabetes medication. Ultimately, 1024 eligible participants were included in this study (Fig. 1).

### Insulin resistance

Insulin resistance was assessed using the HOMA-IR by the following formula: fasting blood glucose (mmol/L) × serum insulin (µU/mL) /22.5 [22]. Participants who had their insulin and glucose levels measured in the morning were asked to fast the night before. Fasting serum glucose was measured on a Beckman Coulter UniCel^®^ DxC800 Synchron Clinical System [23]. Serum insulin was examined using the AIA-PACK IRI, a two-site immunoenzymometric assay [24]. Detailed testing procedures for blood glucose [25] and insulin [26] are available online.

### Weight-adjusted-waist index

The WWI is an anthropometric index for assessing obesity that evaluates body fat mass and muscle mass by normalizing WC for weight and is a new metric of obesity assessment, with higher WWI scores indicating higher degree of obesity [11]. The WWI was calculated by dividing WC (cm) by weight (kg) squared root. To assure the accuracy of the data, trained medical personnel performed anthropometric measurements at a mobile examination center, and professional recorders documented the results. Detailed anthropometric programs are available on the website [27].

### Assessment for physical activity

The data on PA for this study were obtained by using the WHO Global Physical Activity Questionnaire. Adolescents were asked to fill out a questionnaire without parental assistance on their average daily hours of moderate-to-vigorous PA at work, in traffic, and for recreation. Based on their weekly physical activity level, adolescents were divided into three groups: the most active (2 highest quintiles), the second and third quintiles of moderate activity, and the lowest quintile of inactivity [28].

### Covariates

Covariates that might influence the relationship between WWI and physical activity and HOMA-IR were taken into account in our study [14, 29], including age, sex (male or female), race (Mexican American, other Hispanic, non-Hispanic White, non-Hispanic Black, other race), family income to poverty ratio (PIR) (< 1 or ≥ 1), secondhand smoke status (yes or no), and survey cycle (2013–2014 and 2015–2016). Secondhand smoke was defined as self-reported exposure to burning cigarettes or smoke exhaled by smokers, or serum cotinine levels ≥ 0.05 ng/mL [30].

### Statistical analysis

Descriptive analyses were conducted for baseline characteristics of participants, categorical variables are characterized by frequency (percentage), whereas continuous variables are represented by averages with a standard deviation (Mean ± SD) and/or median (25th, 75th). Since the HOMA-IR is skewed, it is corrected for this using the natural logarithm (ln) transformation.

The associations of WWI and PA with HOMA-IR were estimated using linear regression model. To further assess the association between WWI and HOMA-IR, WWI was included in the linear regression model by dividing WWI into quartiles, and *P* trend was calculated by fitting the median of WWI quartile to a continuous variable. Moreover, generalized additive models and smooth curve fittings were applied to investigate the nonlinear relationships. The adjustment model adjusted for variables such as age, sex, race, family PIR, secondhand smoke status, and survey cycle.

The combined associations of the WWI and PA with HOMA-IR were further evaluated. Participants were divided into low and high WWI groups based on the median values of WWI. In addition, participants were divided into inactive, and active groups based on the PA. The group with low WWI and inactive was considered as the reference group. Subgroup analyses were performed by sex, while adjusting for the covariates described above.

All statistical analyses were conducted using R (version 4.2.2), and *P* < 0.05 was considered with statistically significant.

## Results

### Baseline characteristics

A total of 1,024 participants were included in this study, and the characteristics of the participants are listed in Table 1. The median age of the participants was 15 (13, 17) years. The distribution of sex was relatively balanced with 50.8% males and 49.2% females. Of the study population, 26.5% were non-Hispanic White, 706 (68.9%) were at or above the poverty level, 491 (47.9%) were exposed to secondhand smoking, and 214 (20.8%) were physically inactive. The median of WWI and HOMA-IR were 10.10 (9.56, 10.64) and 2.27 (1.50, 3.62), respectively.

**Table 1:** Characteristics of included participants from NHANES 2013–2016 (*N* = 1,024)

Characteristics	*N* (%)/Median (25th, 75th)
Age (years)	15 (13, 17)
Sex	
Male	520 (50.8)
Female	504 (49.2)
Race	
Mexican American	234 (22.9)
Other Hispanic	101 (9.9)
Non-Hispanic White	272 (26.5)
Non-Hispanic Black	262 (25.6)
Other race	155 (15.1)
Family PIR	
< 1	318 (31.1)
≥ 1	706 (68.9)
Second hand smoking	
Yes	491 (47.9)
No	533 (52.1)
Survey cycle	
2013–2014	543 (53.0)
2015–2016	481 (47.0)
Physical activity (MET min/week)	
Inactive (< 400)	214 (20.8)
Moderately active (400–2880)	397 (38.5)
Active (≥ 2880)	420 (40.7)
Body mass index (kg/m^2^)	
Under weight	23 (2.2)
Normal weight	600 (58.6)
Over weight	169 (16.5)
Obesity	232 (22.7)

Abbreviations: NHANES, National health and Nutrition Examination Survey; PIR, HOMA-IR, Homeostatic Model Assessment of insulin resistance; Family PIR, Family income to poverty ratio

### Distribution of anthropometric indicators and HOMA-IR

The levels of WWI, WC, and HOMA-IR in the study population are shown in Table S1. The median and geometric mean of WWI were 10.098 (9.562, 10.644) and 10.125, respectively. And the median and geometric mean of WC (cm) were 78.400 (71.200, 89.200) and 81.015, respectively. The median HOMA-IR was 2.274 (1.500, 3.623), as well as the geometric mean was 2.403.

### The association of WWI and HOMA-IR

**Fig. 2:**
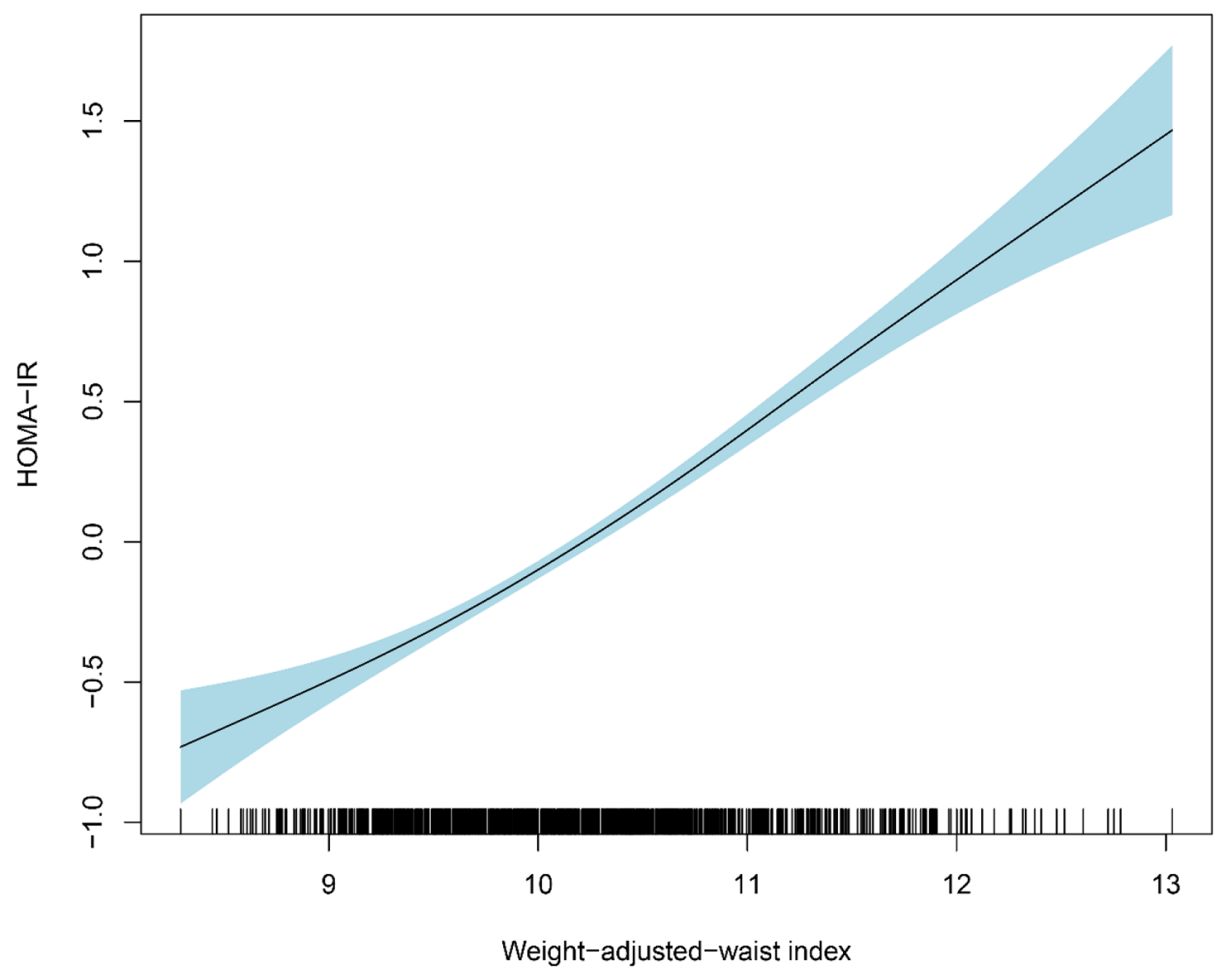
Smooth curve fitting for WWI and HOMA-IR. Non-linear relationship between WWI and HOMA-IR was detected by the generalized additive model. The solid black line represents the smooth curve fit between variables. The shaded area represents the 95% CI from the fit

The relationship between WWI and ln-HOMA-IR is presented in Table 2. A significant positive association between WWI and ln-HOMA-IR was observed in both the adjusted (β: 0.48; 95% CI: 0.43, 0.53) and unadjusted (β: 0.44; 95% CI: 0.40, 0.49) models. This positive association remained stable after the WWI was transformed into quartiles. According to the adjusted models, elevated ln-HOMA-IR was observed in subjects with the second (β: 0.23; 95% CI: 0.12, 0.34), third (β: 0.47; 95% CI: 0.35, 0.59), and highest (β: 0.95; 95% CI: 0.83, 1.06) quartile of WWI compared with those with the lowest quartile with (*P* for trend < 0.05). Moreover, a nonlinear positive association between WWI and ln-HOMA-IR was observed according to the results of the analysis of smoothed curve fitting (Fig. 2).

**Table 2:** Associations between weight-adjusted-waist index and HOMA-IR.

WWI	Unadjusted model β (95% CI)	*P* value	Adjusted model β (95% CI)	*P* value
Continuous	0.44 (0.40, 0.49)	< 0.001	0.48 (0.43, 0.53)	< 0.001
Quartile				
Q1 (< 9.56)	Reference		Reference	
Q2 (9.56–10.10)	0.20 (0.09, 0.30)	< 0.001	0.23 (0.12, 0.34)	< 0.001
Q3 (10.10-10.64)	0.41 (0.30, 0.52)	< 0.001	0.47 (0.35, 0.59)	< 0.001
Q4 (≥ 10.64)	0.91 (0.80, 1.01)	< 0.001	0.95 (0.83, 1.06)	< 0.001
*P* for trend	< 0.001		< 0.001	

Abbreviation: CI, confidence interval; HOMA-IR, Homeostatic Model Assessment of insulin resistance; WWI, weight-adjusted-waist index

Adjusted for age, sex, race, family income to poverty ratio, and survey cycle. second hand smoking, and physical activity

*P* for trend across quartiles of weight-adjusted-waist index

### The association of PA and HOMA-IR

The correlation between PA and ln-HOMA-IR was displayed in Table 3. After adjusting for covariates, the ln-HOMA-IR was lower in participants who were moderately physically active (β: -0.08; 95% CI: -0.17, 0.02) or physically active (β: -0.16; 95% CI: -0.26, -0.05), compared with those who were inactive. In addition, similar associations were observed in the unadjusted models.

**Table 3:** Associations between physical activity and HOMA-IR.

Physical activity	Unadjusted model β (95% CI)	*P* value	Adjusted model β (95% CI)	*P* value
Inactive	Reference		Reference	
Moderately active	-0.16 (-0.27, -0.04)	0.008	-0.08 (-0.17, 0.02)	0.133
Active	-0.33 (-0.44, -0.21)	< 0.001	-0.16 (-0.26, -0.05)	0.003

Abbreviation: CI, confidence interval; HOMA-IR, Homeostatic Model Assessment of insulin resistance; WWI, weight-adjusted-waist index

Adjusted for age, sex, race, family income to poverty ratio, survey cycle. second hand smoking, and weight-adjusted-waist index

### The association of waist circumference and HOMA-IR

As shown in Table S2, WC was positively associated with ln-HOMA-IR (β: 0.029; 95% CI: 0.027, 0.031) after adjusting for covariates. With increasing quartiles of WC, the ln-HOMA-IR was increased in a dose-dependent manner. The relationships between WC and ln-HOMA-IR were not substantially affected by adjusting for possible confounders [β (95%CIs): Q1 = reference; Q2 = 0.108 (0.010, 0.206); Q3 = 0.377 (0.279, 0.475); Q4 = 1.053 (0.953, 1.152)].

### Joint association between WWI and PA and HOMA-IR

Regarding the joint association of WWI and PA with ln-HOMA-IR (Table 4), participants who had lower WWI and were not physically active (β: 0.21; 95% CI: 0.05, 0.36), participants who had higher WWI and were physically active (β: 0.59; 95% CI: 0.49, 0.68), and participants who had higher WWI and were not physically active (β: 0.69; 95% CI: 0.56, 0.82) all had an elevated ln-HOMA-IR compared to participants who had lower WWI and were physically active. Moreover, participants who had higher WWI and were not physically active had the greatest elevation in ln-HOMA-IR, which revealed a joint association of WWI with PA.

**Table 4:** The joint association of weight-adjusted-waist index and physical activity on HOMA-IR.

WWI- PA	*N*	Unadjusted model β (95% CI)	Adjusted model β (95% CI)
Low WWI + Active	436	Reference	Reference
Low WWI + Inactive	76	0.21 (0.05, 0.36)	0.21 (0.05, 0.36)
High WWI + Active	375	0.56 (0.48, 0.65)	0.59 (0.49, 0.68)
High WWI + Inactive	137	0.67 (0.55, 0.79)	0.69 (0.56, 0.82)

Abbreviation: CI, confidence interval; PA, physical activity; HOMA-IR, Homeostatic Model Assessment of insulin resistance; WWI, weight-adjusted-waist index

Adjusted for age, sex, race, family income to poverty ratio, second hand smoking, and survey cycle

### Subgroup analyses

To test the consistency of the relationship between WWI and HOMA-IR in different populations, we performed subgroup analyses stratified by sex. The findings indicate a consistent association. A significant positive association between WWI and HOMA-IR was found in both males (β: 0.52; 95% CI: 0.45, 0.59) and females (β: 0.43; 95% CI: 0.36, 0.51) (Table S3). The HOMA-IR was lower in both males and females who were moderately physically active or physically active, compared with those who were inactive, although the association was not significant in females (Table S4). Moreover, males and females with higher WWI and were not physically active had the greatest elevation of HOMA-IR, suggesting a positive combined association of WWI and PA (Table S5). Furthermore, the findings of smoothed curve fitting demonstrated a nonlinear positive correlation between WWI and HOMA-IR in males and females (Fig. S1).

## Discussion

This cross-sectional study involving 1,024 adolescents was designed to evaluate the relationship between WWI and PA and HOMA-IR, as well as to assess the joint association of WWI and PA on HOMA-IR in US adolescents. We observed a significant positive association between WWI and HOMA-IR, and a significant negative association between PA and HOMA-IR. This significant correlation remained even after categorizing WWI into quartiles (Q1-Q4). In addition, we observed a joint association of WWI and PA on the HOMA-IR. These observations suggest that WWI and PA are independently associated with HOMA-IR, which provides an important reference in the prevention and management of HOMA-IR in adolescents.

As far as we are aware, this is the first research to examine the association between WWI and HOMA-IR and to explore the joint association of WWI and PA. BMI and WC are internationally recognized as the two main indicators of obesity. Numerous studies have shown that BMI is positively correlated with HOMA-IR [31–33]. Additionally, the study discovered that that in patients with idiopathic hypogonadism, normal glucose tolerance, and normal body weight, body fat mass was an independent predictor of insulin resistance [29]. In the normoglycemic population, higher HOMA-IR values were associated with higher WC values [34]. Another study showed a significant positive association between WC and HOMA-IR [35]. Despite being a widely used anthropometric measure, BMI cannot distinguish between adipose and lean tissue mass. Some research has shown that adding body mass index to models could enhance the prediction of abdominal subcutaneous fat mass by WC due to WC is frequently used to indirectly assess visceral fat [36]. However, more extensive clinical research and practical applications are required to validate the accuracy of these indicators. An increasing number of relevant studies have demonstrated the potential of the WWI as a novel index of obesity in recent years. In Korea, a nationwide comprehensive cohort research with 465,629 people found that WWI was a better predictor of cardiometabolic disease and death than BMI, WC, and waist-to-hip ratio [11]. Recent studies have also found that WWI is the strongest predictor of a wide range of other diseases, surpassing BMI and WC [37, 38]. Thus, WWI may assess obesity more comprehensively and accurately and reflect the relationship between obesity and HOMA-IR more accurately.

Considering that IR is a significant risk factor for the onset of cardiovascular disease, understanding the mechanisms that lead to IR is critical to identify populations in children and adolescents with obesity that deserve special attention. An important etiology of insulin resistance in people with obesity is hyperlipidemia [39]. Inflammation of the adipose tissue appears to exacerbate insulin resistance in other insulin-sensitive organs by elevating the concentration of free fatty acids in the blood [39]. In addition, central obesity increases oxidative stress in the body, and oxidative stress can induce IR by impairing insulin signaling and causing dysregulation of adipokines [40]. The release of reactive oxygen species from adipose tissue is increased in people with obesity [41]. Additional variables have been suggested as contributing to the pathogenesis of obesity-induced IR, such as decreased β-oxidation, mitochondrial dysfunction, and intracellular lipid buildup in the liver and skeletal muscle [42].

Our findings demonstrated an inverse association between increased PA and HOMA-IR, which is consistent with previous studies [43]. Previous studies have also found that increased PA may have an independent effect on improving insulin sensitivity [44, 45]. Despite the fact that a large body of research demonstrates the positive impact of PA on insulin resistance, it is still unclear whether the benefits of exercise stem from the reduction in WC or from the exercise itself [46, 47]. A cross-sectional study of U.S. adults demonstrated that PA was associated with IR [47]. The link between PA levels and HOMA-IR may be mediated by visceral fat expressed as WC, as this relationship vanished after WC was taken into account. After correcting for WC, another study conducted on a Canadian population revealed an independent relationship between PA and insulin sensitivity in male [48]. A significant negative association between PA and HOMA-IR was found in a study among populations without diabetes, independent of the waist circumference based on the Kangbuk Samsung Health Study [49]. Our study excluded participants with diabetes, which increases the reliability of our results. Moreover, physical activity may reduce HOMA-IR by improving inflammation levels. Kawanishi et al. performed an exercise intervention in rats on a high-fat diet and found that TNF-α with mRNA expression was significantly reduced in the exercise intervention group compared to the no exercise group [50]. It has been found that skeletal muscles secrete large amounts of anti-inflammatory cytokines such as IL-1Ra after prolonged aerobic exercise, which helps to regulate the balance of anti-inflammatory and pro-inflammatory factors in the body [51]. Nonetheless, additional prospective research is necessary to confirm the connection between PA and HOMA-IR.

We further examined the joint association of PA and WWI by dividing participants with different levels of PA and WWI into four groups. This is the first study to assess the combined association of obesity-related indicators and PA on HOMA-IR in adolescents. These findings suggest that both low PA and high WWI are associated with elevated HOMA-IR. Furthermore, the coexistence of low PA and high WWI is associated with higher HOMA-IR compared with either factor alone. This finding is of great significance. In future studies, a more rigorous and comprehensive research design should be employed in order to further confirm and support the initial findings of our study. Moreover, future studies need to progressively refine the boundaries of WWI and PA levels that are conducive to adolescent health, thereby providing more scientific and precise guidance for adolescent health promotion.

The significant strength of this study is that it is the first to examine the correlation between WWI and HOMA-IR in adolescents, with a large and representative sample. In addition, to the best of our knowledge, we evaluated for the first time the combined association of WWI and PA on HOMA-IR in adolescents. Finally, we adjusted for confounders to minimize the effect of confounding factors and obtain more reliable results. Owing to the adolescent population in our study, potential confounding variables, such as pre-existing conditions, occupational exposures, and alcohol intake, had less of an impact on the results.

Given a number of limitations, the conclusions of this study merit cautious examination. Primarily, it is impracticable to establish a causal relationship between WWI and PA and HOMA-IR in adolescents because of the cross-sectional approach used in this study. In addition, there may also be some bias in information collection due to the cross-sectional study design. Moreover, self-reported physical activity information may be somewhat biased. Nonetheless, given feasibility, physical activity is usually assessed through self-reporting in population-based data collection. Finally, even if some possible confounding factors are taken into account, the influence of other factors including alcohol consumption of adolescents cannot be completely ruled out.

## Conclusion

The findings of this study imply that elevated WWI and PA are linked to increased HOMA-IR in adolescents. In addition, WWI and PA have a joint association on HOMA-IR in adolescents. WWI is a new obesity index that standardizes waist circumference to weight. In routine clinical practice, WWI is currently less commonly used to assess obesity and central obesity than BMI and WC. Therefore, more clinical research is necessary to clarify the benefits and drawbacks of WWI.

### Electronic supplementary material

Below is the link to the electronic supplementary material.

Supplementary Material 1

## Data Availability

The data used in this study are publicly available online (https://wwwn.cdc.gov/nchs/nhanes/).
