# Supplementary material for: Joint association of weight-adjusted-waist index and physical activity with insulin resistance in adolescents: a cross-sectional study
Source: BMC Endocr Disord. 2024 Jul 1;24:100. doi: 10.1186/s12902-024-01633-1 (PMC11218192; doi:10.1186/s12902-024-01633-1)
Supplement: Supplementary file 1 — Supplementary Material 1 [file 12902_2024_1633_MOESM1_ESM.docx]

**Joint association of weight-adjusted-waist index and physical activity with insulin resistance in adolescents: A cross-sectional study**

**Table of Contents:**

**Table S1.** Distribution of anthropometric indicators and HOMA-IR.

**Table S2.** Associations between waist circumference and HOMA-IR.

**Table S3:** Associations between WWI and HOMA-IR stratified by sex.

**Table S4:** Associations between physical activity and HOMA-IR stratified by sex.

**Table S5:** The joint effects of weight-adjusted-waist index and physical activity on HOMA-IR stratified by sex.

**Figure of Content:**

**Figure S1:** Smooth curve fitting for WWI and HOMA-IR in male (A) and female (B).

| **Table S1.** Distribution of anthropometric indicators and HOMA-IR. | | | | | | |
| --- | --- | --- | --- | --- | --- | --- |
| Indicators | GM | Percentile | | | | |
|  |  | 5th | 25th | 50th | 75th | 95th |
| WWI | 10.125 | 9.009 | 9.562 | 10.098 | 10.644 | 11.682 |
| WC (cm) | 81.015 | 64.115 | 71.200 | 78.400 | 89.200 | 116.955 |
| HOMA-IR | 2.403 | 0.859 | 1.500 | 2.274 | 3.623 | 8.253 |
| Abbreviation: GM, geometric mean; HOMA-IR, Homeostatic Model Assessment of insulin resistance; WC, waist circumference; WWI, weight-adjusted-waist index. | | | | | | |

| **Table S2.** Associations between waist circumference and HOMA-IR. | | | | |
| --- | --- | --- | --- | --- |
| WC | Unadjusted model  β (95% CI) | *P* value | Adjusted model  β (95% CI) | *P* value |
| Continuous | 0.027 (0.025, 0.029) | < 0.001 | 0.029 (0.027, 0.031) | < 0.001 |
| Quartile |  |  |  |  |
| Q1 (< 71.20) | Reference |  | Reference |  |
| Q2 (71.20-78.40) | 0.011 (-0.089, 0.112) | < 0.827 | 0.108 (0.010, 0.206) | 0.031 |
| Q3 (78.40-89.20) | 0.296 (0.195, 0.396) | < 0.001 | 0.377 (0.279, 0.475) | < 0.001 |
| Q4 (≥ 89.20) | 0.966 (0.866, 1.087) | < 0.001 | 1.053 (0.953, 1.152) | < 0.001 |
| *P* for trend | < 0.001 |  | < 0.001 |  |
| Abbreviation: CI, confidence interval; HOMA-IR, Homeostatic Model Assessment of insulin resistance; WC, waist circumference.  Adjusted for age, sex, race, family income to poverty ratio, and survey cycle. second hand smoking, and physical activity.  *P* for trend across the median values of quartiles of waist circumference. | | | | |

| **Table S3.** Associations between WWI and HOMA-IR stratified by sex. | | | | | |
| --- | --- | --- | --- | --- | --- |
| WWI | Male | |  | Female | |
|  | Unadjusted | Adjusted |  | Unadjusted | Adjusted |
| Continuous | 0.49 (0.43, 0.56) | 0.52 (0.45, 0.59) |  | 0.41 (0.34, 0.48) | 0.43 (0.36, 0.51) |
| Quartile |  |  |  |  |  |
| Q1 | Reference | Reference |  | Reference | Reference |
| Q2 | 0.09 (-0.06, 0.25) | 0.13 (-0.03, 0.28) |  | 0.22 (0.07, 0.37) | 0.29 (0.14, 0.44) |
| Q3 | 0.33 (0.18, 0.48) | 0.35 (0.19, 0.51) |  | 0.24 (0.09, 0.39) | 0.30 (0.15, 0.45) |
| Q4 | 0.99 (0.83, 1.14) | 1.00 (0.84, 1.18) |  | 0.74 (0.59, 0.88) | 0.76 (0.60, 0.91) |
| *P* for trend | < 0.001 | < 0.001 |  | < 0.001 | < 0.001 |
| Abbreviation: CI, confidence interval; HOMA-IR, Homeostatic Model Assessment of insulin resistance; WWI, weight-adjusted-waist index.  Adjusted for age, race, family income to poverty ratio, and survey cycle. second hand smoking, and physical activity.  *P* for trend across the median values of quartiles of weight-adjusted-waist index. | | | | | |

| **Table S4.** Associations between physical activity and HOMA-IR stratified by sex. | | | | | |
| --- | --- | --- | --- | --- | --- |
| WWI | Male | |  | Female | |
|  | Unadjusted | Adjusted |  | Unadjusted | Adjusted |
| Inactive | Reference | Reference |  | Reference | Reference |
| Moderately active | -0.22 (-0.39, -0.05) | -0.12 (-0.26, 0.03) |  | -0.16 (-0.32, -0.01) | -0.11 (-0.25, 0.02) |
| Active | -0.46 (-0.63, -0.29) | -0.24 (-0.39, -0.09) |  | -0.20 (-0.36, -0.05) | -0.11 (-0.25, 0.03) |
| Abbreviation: CI, confidence interval; HOMA-IR, Homeostatic Model Assessment of insulin resistance; WWI, weight-adjusted-waist index.  Adjusted for age, race, family income to poverty ratio, survey cycle. second hand smoking, and weight-adjusted-waist index. | | | | | |

| **Table S5.** The joint associations of weight-adjusted-waist index and physical activity on HOMA-IR stratified by sex. | | | | | |
| --- | --- | --- | --- | --- | --- |
| WWI | Male | |  | Female | |
|  | Unadjusted | Adjusted |  | Unadjusted | Adjusted |
| Low WWI + Active | Reference | Reference |  | Reference | Reference |
| Low WWI + Inactive | 0.21 (-0.03, 0.44) | 0.22 (-0.02, 0.45) |  | 0.11 (-0.11, 0.32) | 0.07 (-0.14, 0.29) |
| High WWI + Active | 0.58 (0.45, 0.70) | 0.59 (0.46, 0.73) |  | 0.37 (0.24, 0.50) | 0.38 (0.25, 0.52) |
| High WWI + Inactive | 0.85 (0.65, 1.01) | 0.84 (0.65, 1.03) |  | 0.50 (0.33, 0.66) | 0.50 (0.33, 0.66) |
| Abbreviation: CI, confidence interval; PA, physical activity; HOMA-IR, Homeostatic Model Assessment of insulin resistance; WWI, weight-adjusted-waist index.  Adjusted for age, race, family income to poverty ratio, second hand smoking, and survey cycle. | | | | | |


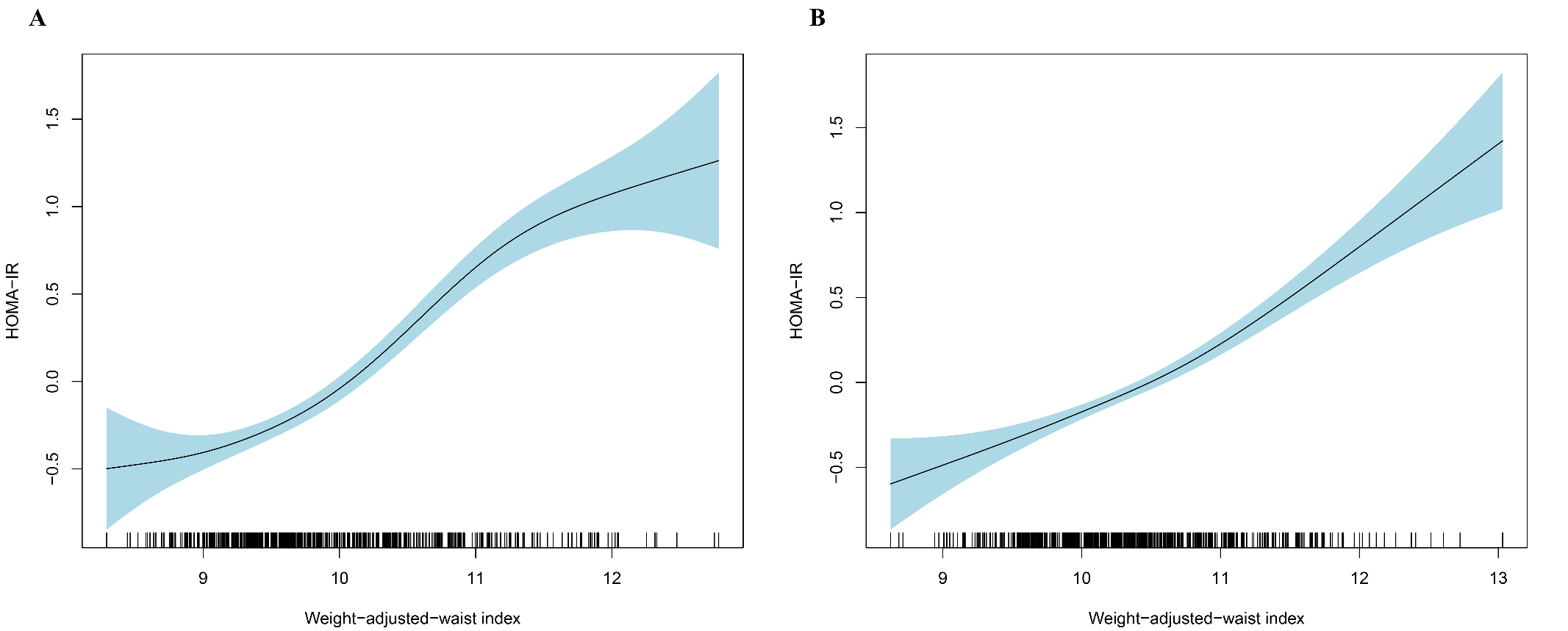


**Figure S1.** Smooth curve fitting for WWI and HOMA-IR in male (A) and female (B). Non-linear relationship between WWI and HOMA-IR was detected by the generalized additive model. The solid black line represents the smooth curve fit between variables. The shaded area represents the 95 % CI from the fit.
